# Sensitivity of Cerebellar Reaching Ataxia to Kinematic and Dynamic Demands

**DOI:** 10.1101/2024.10.28.620711

**Published:** 2024-10-30

**Authors:** Kyunggeune Oh, Di Cao, Noah Cowan, Amy Bastian

**Affiliations:** a. Center for Movement studies, Kennedy Krieger Institute, Baltimore, Maryland; b. Department of Neuroscience, Johns Hopkins University, Baltimore, Maryland; c. Department of Mechanical Engineering, Johns Hopkins University, Baltimore, Maryland; d. Laboratory for Computational Sensing and Robotics, Johns Hopkins University, Baltimore, Maryland

## Abstract

Individuals with cerebellar ataxia often face significant challenges in controlling reaching, especially when multijoint movements are involved. This study investigated the effects of kinematic and dynamic demands on reaching movements by individuals with cerebellar ataxia and healthy controls using a virtual reality task. Participants reached to target locations designed to elicit a range of coordination strategies between shoulder and elbow joint movements. Results showed that the cerebellar group exhibited greater trajectory curvature and variability in hand paths compared to controls, with pronounced deficits in the initial hand movement direction. Kinematic simulations indicated that early hand movement errors were sensitive to the required onset times and rates of joint movements and were most impaired when opposite direction joint movements were required (e.g., elbow extension with shoulder flexion). This highlights significant disruptions in motion planning and feedforward control in the cerebellar group. Dynamic analysis showed that cerebellar participants’ movements were more impaired in reaching directions where interaction torques normally assist the desired elbow and shoulder movements, which required them to rely more on muscle torques to move. These reach directions were also those that required opposite direction joint movements. Overall, our data suggest that reaching deficits in cerebellar ataxia result from 1) the early-phase motion planning deficits that worsen with tight timing requirements and 2) the inability to compensate for interaction torques, particularly when they assist the intended movement.

## Introduction

A hallmark of cerebellar damage is movement incoordination (ataxia). For example, people with ataxia reach with hand paths that show abnormal curvature and oscillation as they home in on a target. Many studies have quantified kinematic abnormalities in an effort to understand the fundamental motor control deficits associated with reaching ataxia. Some studies have used reductionist methods by physically constraining movement to a single-joint (e.g., elbow) and show kinematic abnormalities including some dysmetria (i.e. over- or undershooting), oscillation and prolonged deceleration phases (Bhanpuri et al. 2014; Hallett and Massaquoi 1993; Zimmet et al. 2020). Several groups have reported that multijointed reaches are more impaired than single-jointed reaches, due to abnormal coordination between joints (Bastian and Thach 1995; Goodkin et al. 1993; Thach et al. 1992). Consistent with this, it has been found that a single jointed elbow movement worsens when it is made without shoulder constraint, due to poor shoulder stabilization (Bastian et al. 2000). These findings suggest that coordination problems associated with cerebellar ataxia may stem from poor control of the more complicated dynamics of multijointed movements.

One hypothesis is that ataxia may be due to poor control of specific elements of movement dynamics. People with cerebellar ataxia have been reported to have difficulty predicting and accounting for "interaction torques”, which are produced at a given joint (e.g. elbow) by motions of other linked joints (e.g. shoulder) (Bastian et al. 1996; Topka et al. 1998a; Topka et al. 1998b). Interaction torques scale with movement velocity and can induce large joint accelerations, powerfully affecting reaching trajectories. Accordingly, when people with cerebellar ataxia make faster reaching movements, they show greater overshooting errors due to poor compensation for interaction torques (Bastian et al. 1996; Topka et al. 1998a; Topka et al. 1998b). Interaction torques also exhibit non-linear characteristics, depending on the number of joints involved, their initial configurations, and relative movement directions (Bastian et al. 2000; Gribble and Ostry 1999; Hollerbach and Flash 1982). Importantly, depending on the reaching direction, interaction torques can assist or resist desired joint motions (Galloway and Koshland 2002). Typical adults can predictively utilize or counteract interaction torques to make a well-coordinated reach (Maeda et al. 2017). It is not clear if people with cerebellar ataxia show different patterns of deficit depending on whether interaction torques assist or resist the desired joint motions in reaching.

Impaired dynamic control may explain why people with cerebellar damage show errors early in reaching that vary with movement direction (Germanotta et al. 2015; Gibo et al. 2013; Maschke et al. 2004; Zimmet et al. 2019). This has been observed in several studies that used a “center-out” reaching tasks in the horizontal plane, with equidistant targets from a central starting point, like a clock face. Using this task, people with ataxia can make early reaching errors rotated clockwise or counterclockwise relative to the desired target (e.g., Gibo et al. 2013). Each of the aforementioned studies noted that some reaching directions appeared “easier” than others—people with cerebellar damage could reach directly to some targets but showed large errors for other targets. It is difficult to find a systematic pattern across these studies because they used different initial starting positions, different numbers and positions of targets and involved different robots that altered the inertia of the arm.

Here we were interested in understanding if people with cerebellar ataxia show different sensitivities to the kinematic and dynamic demands of reaches made with different combinations of joint movements. We used virtual reality to display reaching targets that were classified according to the direction and combination of standard 20-degree shoulder and 20-degree elbow joint motions. Some combinations required tight control of the timing of shoulder and elbow kinematics for reaching, whereas others did not. In addition, some combinations involved interaction torques that assisted the desired joint motions for reaching, whereas others involved resistive interaction torques. Although interaction torques can be accentuated by fast movements, facilitating analysis (Bastian et al. 1996; Bastian et al. 2000; Becker et al. 1991; Haggard et al. 1994; Topka et al. 1998a; Topka et al. 1998b), natural arm reaching speeds can nevertheless elicit them (Bhanpuri et al. 2014; Hallett and Massaquoi 1993; Zimmet et al. 2020). Thus, we focus here on reaching movements that were at natural and comfortable speeds, as such movements may provide the most insight into everyday activities performed by individuals with cerebellar ataxia.

## Materials and methods

### Subjects

Seventeen healthy control subjects and sixteen individuals with cerebellar ataxia were enrolled in this study. All participants were right-handed, and the control subjects were matched to the cerebellar ataxia subjects in terms of age and sex, as detailed in Table 1. The Scale for the Assessment and Rating of Ataxia (SARA) was employed to evaluate the severity of ataxia in all cerebellar patients. The assessments were conducted remotely via Zoom video calls (Zoom Video Communications, Inc., San Jose, CA, USA), utilizing dual-camera angles: one from the laptop's built-in camera facing the subjects and another from a webcam positioned to their right side. Note that remote administration of the SARA has been shown to be valid and reliable (Reoli et al. 2024). Subjects with severe ataxia were accompanied by a caregiver during the assessment. A summary of the cerebellar subjects' information can be found in Table 2. Informed consent was secured from all participants in this study.

### Apparatus

We designed a remote data collection system that was delivered to each participant’s home, during the COVID-19 pandemic. This allowed us to study people with cerebellar ataxia who lived far away from the lab—our subjects were located in Maryland, Virginia, Vermont, North Carolina Tennessee, Florida, Illinois, Missouri and California. The data collection equipment package included an Oculus Rift S virtual reality (VR) device (Meta Reality Labs, WA, USA) for hand movement measure, a Logitech C922 webcam for monitoring subjects’ sagittal view, and a gaming laptop for video call and data collection. Participants received a detailed set of instructions on how to connect all equipment with color coded cables and photos. After the computer was connected to the internet, the remaining equipment set up was guided by investigators through a video call and remote control of the laptop using TeamViewer (Google LLC, CA, USA). During testing, subjects sat in a stable chair with their feet on the floor wearing the Oculus Rift. The laptop sat on a stable surface in front of them and the webcam was positioned to capture a view of their right side in the sagittal plane. Control participants were tested either off- or on-site. If on-site, the subjects were not involved in setting up the devices. Otherwise, all testing procedures were identical.

### Subject calibration

Before beginning the reaching study, each subject was asked to have another person measure lengths of their arm segments, including the upper arm segment (from the acromion to radiale) and the lower arm and hand segment (from radiale to proximal interphalangeal joint of the index finger). This was done while we were online with the subject, and we provided verbal instructions and video check. The subject was then instructed to sit on a chair and maintain a still trunk and head position during the task either by leaning on the back of the chair or sitting upright.

In the initial stage of the VR task, all subjects underwent a calibration process. They put on the VR headset and held the VR controllers in their hands and were told that the green arrows marked on the floor pointed in the direction that they should be facing. After verifying that they were facing the correct direction, they were asked to raise both their hands VR controllers to the height of their shoulder and extend them forward shoulder-width apart. The position of the right shoulder in 3D space was estimated by recording the positions of the controllers and utilizing the measured lengths of the arm segments.

### Paradigm

**Figure 1.**
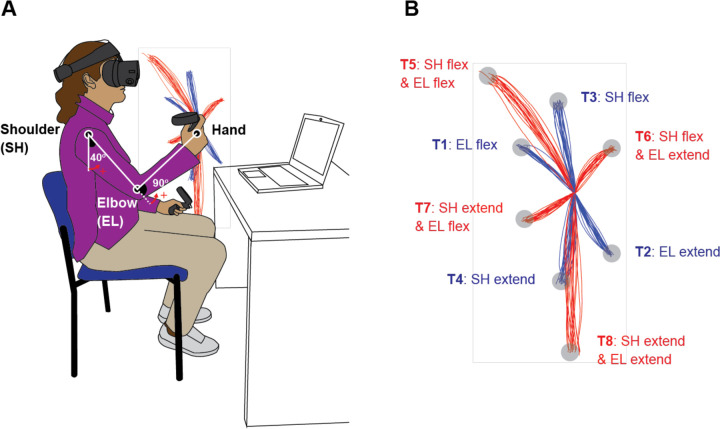
Task Diagram. A. Initial Position: The shoulder joint is angled at 40 degrees from the ground direction, and the elbow joint is at 90 degrees, positioning the hand at the height of the right shoulder. The positive direction for the joint angles is counterclockwise when viewed from the right. B. Target Locations: The starting point and all targets are located on a sagittal plane that aligns with the right shoulder. Hand paths leading to four single-joint targets are illustrated in red, while those leading to four double-joint targets are indicated in blue.

Subjects performed goal-directed arm reaching movements in the sagittal plane in front of their right shoulders. The starting position of the arm reach was defined with the elbow joint angle at 90 degrees and the shoulder joint angle at 40 degrees relative to the ground, as shown in Figure 1. Reaches were performed to eight targets that were displayed one at a time during the task. Targets were defined in the vertical plane, with four of them being single-joint targets that could be reached with 20 degrees of excursion in either flexion or extension of the shoulder or elbow joint. The remaining four targets were two-joint targets, requiring 20 degrees of joint excursions in both the shoulder and elbow joints, flexion or extension. A trial was defined as a single reaching movement from the starting position to one of the targets. Before data recording started, the subjects had practice trials until they became accustomed to the reaching task. Participants reached nine times to each of the 8 targets resulting in 72 total trials arranged in a pseudo-random manner.

The task began with a presentation of the starting point indicated by a red sphere with a 2 cm radius with a gray spherical polygon frame surrounding it. The subject’s hand (the right VR controller) position was displayed as a white sphere with a 1.5 cm radius. When the subject’s hand was inside the red start point for two seconds, a spherical polygon frame surrounding the start point changed from gray to green. A target then appeared in the sagittal plane and the starting point disappeared. The participant was instructed to reach the targets with their natural arm reaching speed and go straight to the targets. All the targets were 2-cm radius spheres of different colors (other than red) and were surrounded by a spherical polygon frame that changed its color from gray to green if the hand was in the target. After two seconds, the target disappeared, and the starting point was displayed. The task was repeated in this way to display a total of 72 targets. There was no time limit for each reach. The subject took breaks anytime between trials for as long as they wanted. The total task time took about 20~30 mins, and most of subjects took 1~2 breaks that was generally within 5 minutes. The hand movement was recorded by a virtual reality device, Oculus Rift S, with 30Hz sampling rate. The joint motions of the shoulder and elbow were also monitored by a webcam with a 30Hz frame rate.

### Data analysis

#### Hand paths

Hand position and velocity were lowpass filtered with a 10Hz cut-off frequency using the ‘lowpass’ function of Matlab. A Hampel filter was also applied to the data using ‘hampel’ function of Matlab with a window size 5 to remove potential outlier noise. The onset and offset timings of each trial were defined with 10% of the peak hand velocity.

We calculated kinematic parameters within the sagittal plane that reflected different aspects of the reaching movement. The initial direction of the hand was quantified as the angle formed between a straight line from start to target and hand to target at 67 milliseconds (equivalent to 2 data frames) after the onset of the reaching movement. The initial hand direction is a critical measure as it reflects the subjecťs motion planning and the efficiency of their feedforward control during the early phase of the reaching movement (Alhussein and Smith 2021; Haith et al. 2015). The maximum deviation ratio was used to represent curvature of the hand path. It was calculated as the perpendicular distance from a line connecting the start and end target to the farthest deviation of the hand path. This value was normalized to the distance from start to end target. A path length ratio was used to evaluate the kinematic efficiency of the hand movement. It was calculated by dividing the actual distance traveled by the hand by the straight-line distance between the start and the end target. Efficient kinematics (lower path ratio) refers to movements that go straight to the target, and inefficient kinematics (higher path ratio) could follow a circuitous or oscillatory path to the target. Lastly, peak hand path velocities, as well as acceleration and deceleration times, were also calculated.

#### Joint angles

Joint angles were calculated using inverse kinematics assuming purely sagittal plane motion (note that the average maximum out of plane motion was small, 0.78 cm for healthy control group and 1.94 cm for cerebellar ataxia group). The participants’ right shoulder was set as the origin of the coordinate system, with the anterior (+) – posterior (−) direction as the x-axis and the upward (+) – downward (−) direction as the y-axis. The hand position was denoted (X,Y). Using the given arm segment lengths L1 (upper arm) and L2 (lower arm and hand), the shoulder $\left(\theta_{SH}\right)$ and elbow $\left(\theta_{EL}\right)$ joint angles were calculated as follows:

(1) $$\Theta_{EL}=\text{acos}\left(\frac{X^{2}+Y^{2}-L1^{2}-L2^{2}}{2*L1*L2}\right)$$

(2) $$\Theta_{SH}=\text{atan}\left(\frac{Y}{X}\right)-\text{atan}\left(\frac{L2*\sin\Theta_{EL}}{L1+L2*\cos\Theta_{EL}}\right)$$

The elbow and shoulder angles are depicted in Figure 1A.

#### Kinematic simulation

A kinematic simulation was conducted to examine the impact of coordination between the shoulder and elbow joints on hand movement during reaching movements toward two-joint targets. In the first step of the simulation, individual mean trajectories of shoulder and elbow joint angles were calculated for each healthy control subject, followed by time normalization so that reach time was expressed from 0–100%. This allowed us to create average joint motions in the two groups. Notably, among healthy control subjects, there was minimal variation in total reach time and joint trajectory shape (Figures 4A and 5A). Subsequently, the all-subject mean curves for shoulder and elbow joint angles were subjected to sigmoidal curve fitting (Equation 3) to calculate the amplitude (a), slope (r), and center point (c).


(3) $$y=\frac{a}{1+e^{\left(-\left(t-c\right)*\frac{4r}{\left|a\right|}\right)}}$$

The simulation was conducted to assess the effect of joint coordination on early hand motion by artificially altering the relative onset times and slopes (i.e. rates) of joint movements. We then simulated how much the hand deviated from the target direction for all combinations of simulated joint motions (Figure 6). The hand deviation angle was calculated at the 25% point of the total reaching time because it provided a good estimate of the rate of shoulder and elbow motions in-flight. The initial reach direction was assessed at 67 ms in order to reflect purely feedforward control.

#### Joint torque

Joint torques were calculated using inverse dynamics equations (Bastian et al. 1996), and the calculations were based on estimations of mass and moment of inertia for each segment, (1) upper arm and (2) forearm and hand (Winter 1990). However, as wrist movement was restricted in this study, wrist angle, angular velocity, and angular acceleration were all set to zero. The inverse dynamics calculations produced net torque (NET), gravity torque (GRAV), muscle torque (MUS), and interaction torque (INT). The basic torque equation used is NET = MUS - INT - GRAV. However, to capture the dynamic variations in the muscle torque components, we calculated the dynamic muscle torque (DMUS= MUS-GRAV), which represents the residual torque after subtracting the gravitational component (Yamasaki et al. 2008). To evaluate the relationship between dynamic muscle torque and interaction torque, we computed the zero-lag cross-correlation between DMUS and INT.

To assess the relative contributions of DMUS and INT to NET, we calculated the contribution index (Sainburg and Kalakanis 2000; Vu et al. 2016; Yamasaki et al. 2008). First, it is essential to recognize that torque impulse represents the integral of torque over a given time interval. We begin by analyzing the contribution of INT to NET. Time intervals during which INT aligned with the direction of the NET were considered to contribute a positive interaction torque impulse, whereas intervals where INT opposed the NET were classified as contributing to a negative interaction torque impulse. The total interaction torque impulse, obtained by summing both positive and negative impulses over the movement duration, was divided by the absolute net impulse to calculate the contribution index of INT to NET. Similarly, the DMUS impulse was computed as its contribution to the NET over the entire movement duration. The sum of the INT and DMUS indexes is always 1.

### Statistical analysis

A two-way ANOVA was conducted to examine the effects of the between-subjects factor (group: healthy controls and cerebellar patients) and the within-subjects factor (target). For post hoc comparisons, a Bonferroni correction was applied. When we examine the correlations between variables, Pearson's correlation coefficient was employed. To ensure the robustness of this approach, we checked for homogeneity of variances between the variables using Levene's test.

## Results

### Hand kinematic analysis

**Figure 2.**
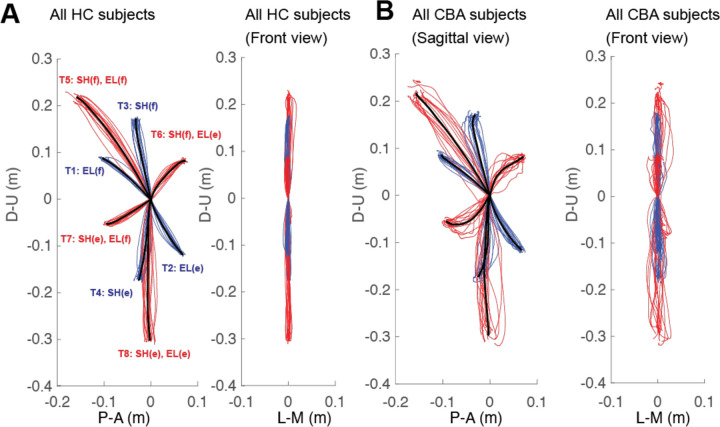
Hand paths. A. Mean hand path trajectories of all healthy control subjects (left: sagittal view from the right; right: front view). B. Mean hand path trajectories of all cerebellar ataxia subjects (left: sagittal view from the right; right: front view). Individual lines represent the mean curves of each subject, and the thick black lines denote the mean trajectories of each subject group. The blue and red lines correspond to hand paths to single-joint and two-joint targets respectively. The hand trajectories of the different subjects were scaled to the average target distances of all subjects. Axes display directions: Y-axis (up-down) and X-axis (anterior-posterior for sagittal view, medial-lateral for front view). Units are in meters.

Figure 2 exhibits the arm reaching hand paths of the control group and the cerebellar group. A thin line traces the average hand path curve for each subject, with blue indicating paths to targets reachable via one joint movement, and red indicating those to targets requiring two joint movements. The group average curves are depicted by bold black lines. Since all subjects have target locations adjusted for their arm segment lengths, the hand paths shown in this figure are scaled using the average target positions of each group, with the starting point positioned at the origin.

#### The cerebellar ataxia group exhibited higher inter-subject variation in hand path trajectories.

In Figures 2, a prominent finding is that the hand paths of the cerebellar group exhibit a greater degree of subject-to-subject variation compared to the healthy control group. This aligns with findings from previous research (Day et al. 1998; Therrien et al. 2021). The subject-to-subject variation of the hand paths can be represented by the standard deviation of the maximum deviation (Table 3). Additionally, hand paths viewed from the front show greater variation in the cerebellar group, yet overall, the hand paths of both groups generally remain near the target plane, as evidenced by Figures 2 (maximum out of plane deviation was < 2 cm, see methods).

#### The cerebellar group showed greater hand path curvature compared to controls, and this difference was largest during opposite movements.

Figure 2 shows that the control group's hand paths are relatively straight, while the cerebellar group’s paths are curved. For both groups, the curvature was lowest in single joint movements and was greatest in multijoint movements where one joint flexes while the other extends (and vice versa, targets T6 and T7). We refer to these as opposite movements for simplicity. The cerebellar group showed significantly higher maximum deviation ratio values compared to the control group (F([1,248] = 113.47, p < 0.001), with the difference most pronounced for targets T6 and T7 (F([1,248] = 68.836, Bonferroni post hoc test, p < 0.001; F([1,248] = 91.527, Bonferroni post hoc test, p < 0.001). It is worth recalling that the targets were set based on joint motions—we studied different combinations of 20-degree elbow and/or shoulder joint movements. Therefore, Cartesian measures, such as target distance, may not fully explain the curvature patterns (e.g., short movements are straighter during single-joint movements, but highly curved for opposite movements).

**Figure 3.**
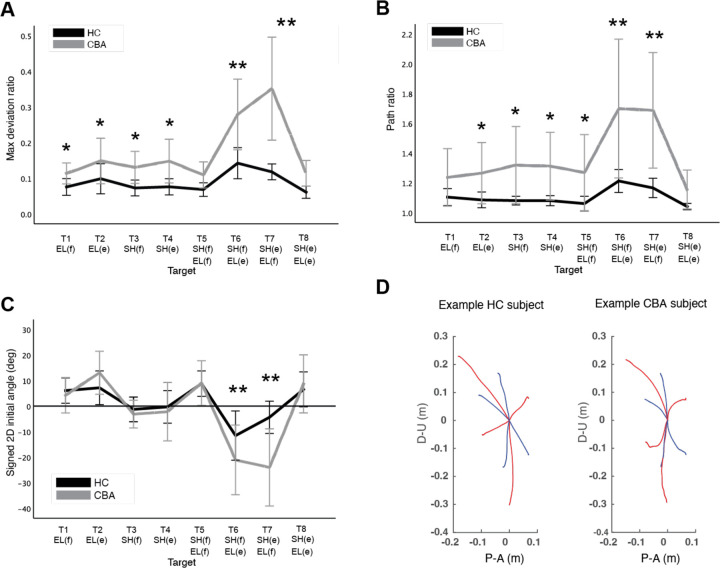
Hand path curvatures. A. Mean maximum deviation ratio. B. Mean path ratio. C. Mean signed 2D initial angle. The sign of hand movement and initial direction was defined relative to the target direction connecting the start point and the target, with counterclockwise considered positive when viewed from the right side of the participants. *: p<0.05, **: p<0.001. D. Mean hand path trajectories of representative healthy control and cerebellar ataxia subjects. The Y-axis represents upward (positive) and downward (negative) directions, and X-axis represents anterior (positive) and posterior (negative) directions, measured in meter. Blue and red lines represent hand trajectories to single-joint and two-joint targets, respectively.

The second index utilized was the hand path ratio, which represents the relative increase in the actual path distance travelled by the hand compared to the direct distance from the starting point to the target. A perfect straight path would have a hand path ratio of 1; any deviation results in a ratio greater than 1. This metric serves as an indicator of the hand path curvature and is also interpretable as a measure of the efficiency of the reaching movement. The results, depicted in Figure 3B, demonstrate that for most targets, cerebellar patients' hand paths were measured to be longer compared to those of healthy control subjects (F[1, 248]=97.754, p<0.001), with the most substantial differences again observed at targets T6 and T7 (F([1,248] = 45.091, Bonferroni post hoc test, p < 0.001; F([1,248] = 48.363, Bonferroni post hoc test, p < 0.001).

#### The initial hand movement misdirection was also larger in opposite movements.

Figure 3C shows group data representing the initial hand movement direction. Note that **p**ositive values are clockwise rotations, and negative values are counterclockwise rotations from a line connecting the start to target positions. As demonstrated in Figure 3C, a significant difference in initial hand movement direction was observed only for targets T6 and T7 between groups (F([1,248] = 11.263, Bonferroni post hoc test, p < 0.001; F([1,248] = 41.264, Bonferroni post hoc test, p < 0.001). These targets required coordination of opposite movements. An individual hand path from subjects in each group is illustrated in Figure 3D

#### People with cerebellar ataxia take longer to reach, with similar acceleration times but prolonged deceleration times.

**Figure 4.**
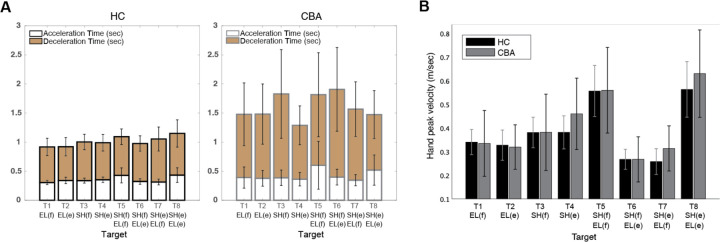
A. Acceleration, deceleration, and total reaching times (sec) for healthy control and cerebellar ataxia groups. B. Peak hand velocity (m/sec) of heathy and CBA groups for each target.

Total reach time, indicated by the entire height of the bar graph in Figure 4A, measures the duration from the onset to the offset of hand movement. The cerebellar group's total reaching time was substantially longer compared to the healthy control group (F[1, 248]=88.176, p<0.001), mainly due to a longer deceleration time. While the average acceleration time—defined as the duration from hand movement onset to peak hand velocity—was 68 milliseconds longer for the cerebellar group (F[7, 248]=1.717, p=0.105, the deceleration time—defined as the period from peak hand velocity to movement offset—exhibited a significant difference of 525 milliseconds (F[7, 248]=5.341, p<0.001).

#### Peak hand velocities were similar between groups.

As indicated in Figure 4B, the peak hand velocity between the two groups was found to be generally similar (F[1, 248]=2.955, p=0.087). In both groups, peak velocity increased proportionally to target distance, consistent with findings from previous goal-directed arm reaching studies (Gordon et al. 1994).

### Arm joint kinematics analysis

#### Joint angle trajectories showed greater inter-subject variation in the cerebellar ataxia group, and this variation was more pronounced in the elbow.

**Figure 5.**
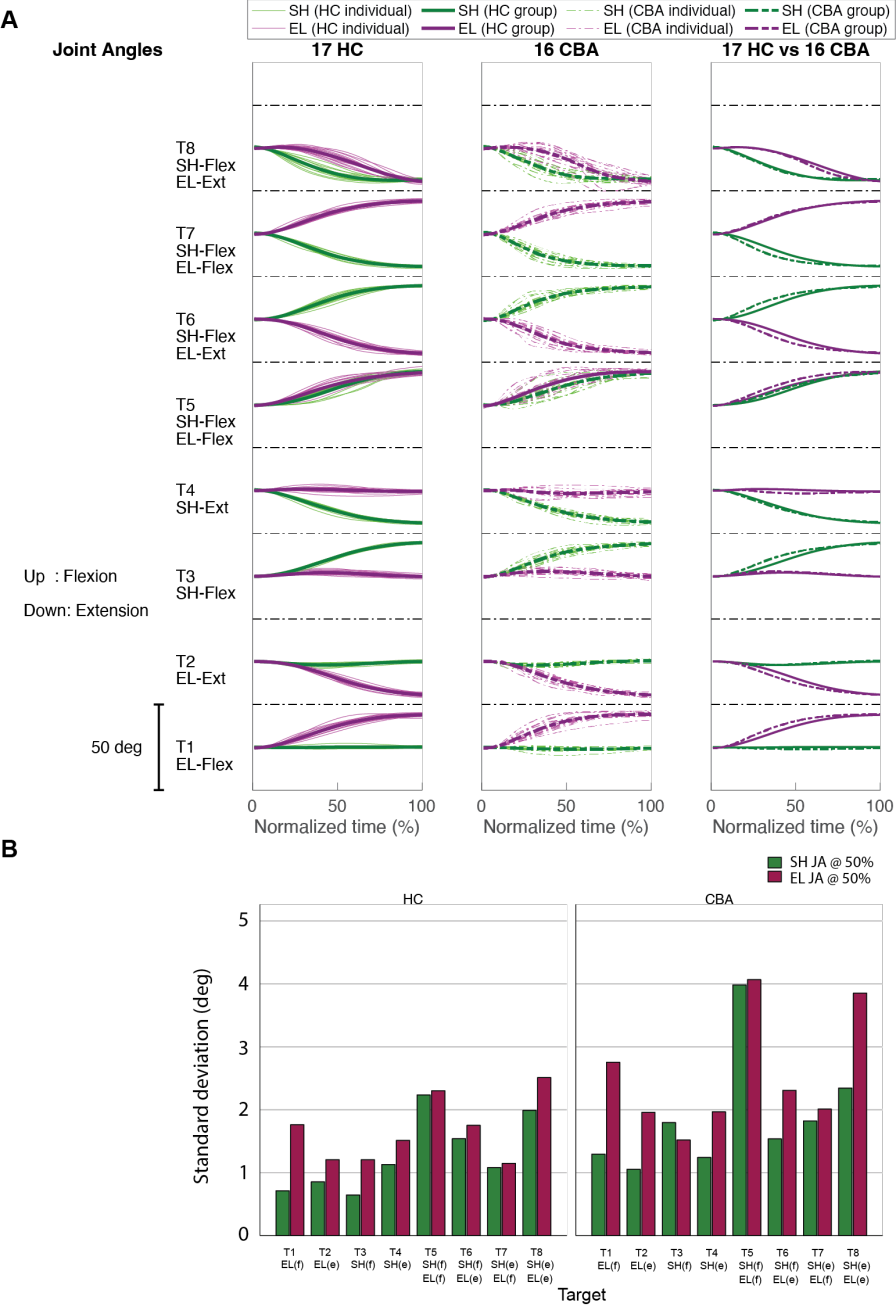
Joint angle trajectories and inter-subject variations. A. Joint angle trajectories for eight target locations, presented in the normalized time. The first and second columns display the individual mean and group mean trajectories for the healthy control and cerebellar group, respectively. Third column only shows group mean trajectories for both groups. B. Joint angle variation (in degree) measured by standard deviation of individual joint angles at 50% of reaching time.

Figure 5A displays the time-normalized joint angle trajectories for all subjects. Time normalization was calculated by setting the arm reaching movement onset as 0% and the offset as 100%. Across the board, the trajectories exhibit a consistent joint excursion of approximately 20 degrees for each joint. However, in terms of trajectory variation, the cerebellar group demonstrates a higher level of variation compared to the healthy control group. When comparing joints, both groups exhibit greater joint angle trajectory variation at the elbow than at the shoulder. The variation in joint angle trajectory was quantified by the standard deviation of the joint angle at the 50% mark on the normalized time axis, with the results depicted in Figure 5B.

### Kinematic simulations of hand deviation for two-joint targets

**Figure 6.**
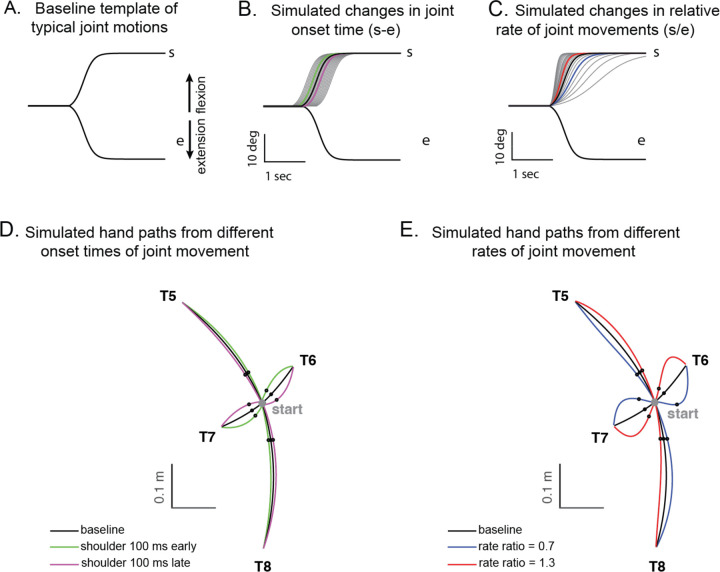
Simulated hand paths resulting from variations in shoulder and elbow joint movements. (A) Baseline template of typical joint motions (mean joint motion of the healthy control group). (B) Simulated changes in shoulder joint onset times. Shoulder joint angle trajectories with onset times 100 msec earlier or later than those of the elbow joint are shown in green and purple, respectively. (C) Simulated changes in the shoulder joint angle rates. The red and blue shoulder angle trajectories represent cases where the relative rate ratio between the shoulder and elbow is 1.3 and 0.7, respectively. (D, E) Joint angle trajectories over time for each condition. The black dots indicate the points where the hand reaches 25% of the distance from the starting position in the direction of the target. At these points, the hand's deviation angle from the target line was calculated.

Kinematic simulations show the sensitivity of hand path deviations to different patterns of shoulder and elbow coordination. Figure 6A–C illustrates how we altered the onset times or rates of shoulder movements relative to a standard elbow movement (examples taken from T6 which required shoulder flexion and elbow extension). The joint trajectories used in this simulation were from a logistic fit to the average joint motions of the control group (see methods). We manipulated shoulder movement to have an earlier or later onset time relative to the elbow (Figure 6B), and a faster or slower rate of movement relative to that of the elbow (Figure 6C). Figures 6D and E show example hand paths resulting from different onset times or relative rates of joint motions for all four multijoint targets (T5-T8.)

**Figure 7.**
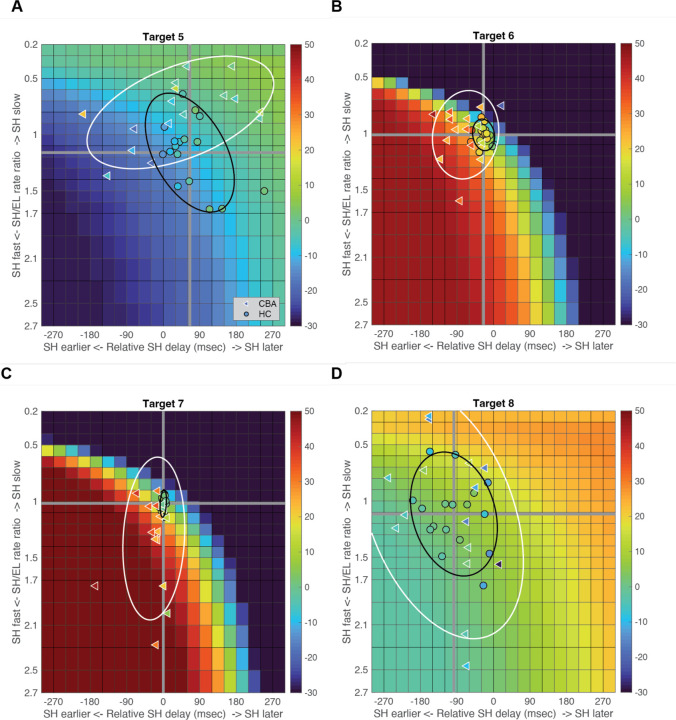
Kinematic simulations of the hand deviation from the target directions for two-joint targets: target 5 (A), 6 (B), 7 (C), and 8 (D). The deviation of hand movement from the target direction at 25% reach time, based on variations in joint onset time and joint change rate (slope) between the shoulder and elbow joints in healthy control subjects. The X-axis shows the onset time difference between the shoulder and elbow joints, where a gray vertical line represents the average onset time difference in healthy controls. The Y-axis indicates the ratio of joint change rates between the shoulder and elbow joints, with a gray horizontal line representing the average rate ratio for the healthy control group. Each subjecťs joint onset time and joint change rate are marked individually, allowing for a comparison between the predicted hand deviation from the simulation and the measured hand deviation from the experiment.

Figure 7 shows the entire colormaps of the kinematic simulations. The color in this matrix represents how much the hand deviates from the target direction. The X-axis shows the onset time difference between the shoulder and elbow joints, and a gray vertical line marks the average onset time difference of healthy control subjects. The Y-axis displays the ratio of joint change rates between the shoulder and elbow joints, with the gray horizontal line representing the average rate ratio for the healthy control group. Movements to Targets 5 and 8, show less sensitivity to relative onset times and rate ratios between joints, as indicated by the gradual shift in color across the maps. In contrast Targets 6 and 7 are highly sensitive, as indicated by the abrupt changes in color across the maps.

We then individually marked each subjecťs joint onset time and joint change rate on the colormap, to compare the hand deviation predicted by the simulation with the hand deviation measured in the experiment. As shown in Table 4, the average difference between the two was 1.69 to 11.56 degrees for the healthy control group and up to 2.62 to 15.79 degrees for the cerebellar ataxia group depending on the target locations, which can be visually confirmed by the color match between the individual data points and the colormap.

Controls tended to show tight control of the joint’s onset times and rate ratios for targets 6 and 7, and looser control for targets 5 and 8. Thus, they appeared to take advantage of reduced precision requirements in joint control when they could. The degree of dispersion between individual subjects’ data was represented by an 80% confidence interval error ellipse. Accordingly, for all four two-joint targets, it was observed that the cerebellar ataxia group's experimental data exhibited generally greater variation in both onset time differences and joint rate ratio differences compared to the healthy control data. For Target 6, the cerebellar group showed a tendency for earlier shoulder onset times, while for Target 7, there was a tendency for a faster relative joint angle change rate in the shoulder.

### Dynamic analysis

#### The torque patterns vary across multijointed targets.

**Figure 8.**
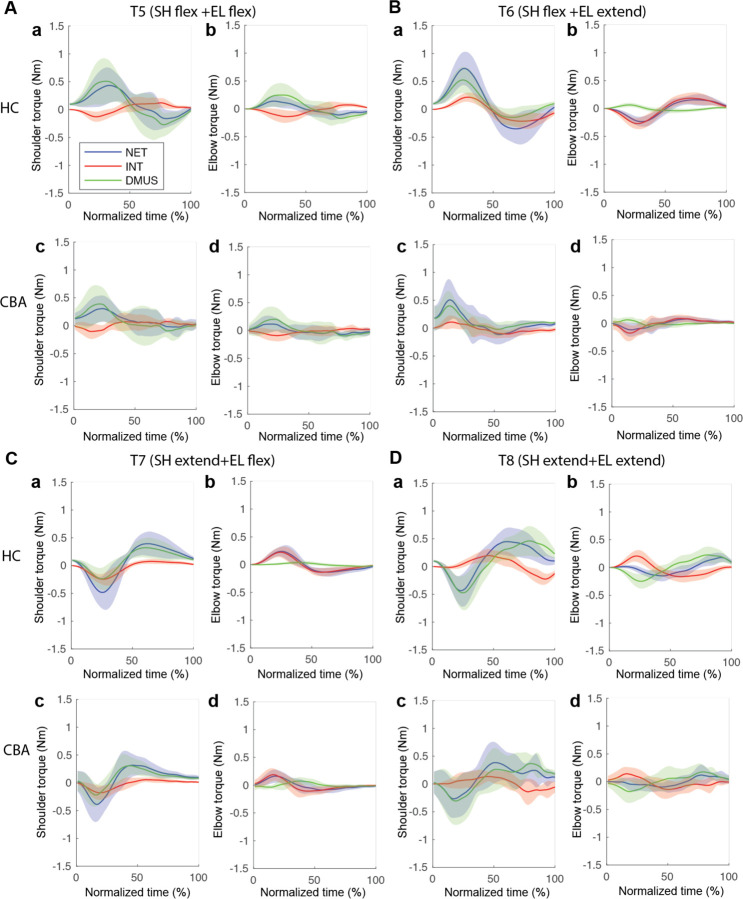
Group mean joint torque for two-joint targets: target 5 (shoulder flexion and elbow flexion, A), target 6 (shoulder flexion and elbow extension, B), target 7 (shoulder extension and elbow flexion, C), and target 8 (shoulder extension and elbow extension, D). Panel a: shoulder joint torques of healthy control group. Panel b: elbow joint torques of healthy control group. Panel c: shoulder joint torques of cerebellar ataxia group. Panel d: elbow joint torques of cerebellar ataxia group.

Figure 8 presents the torque data for four multijoint targets, and illustrates the dynamic interplay between net, interaction, and dynamic muscle torques. As shown in the figure, the arm-reaching movements performed in this study were not very fast, leading to relatively smaller interaction torque compared to net torque and dynamic muscle torque. Additionally, across all four two-joint target movements, net torque and muscle torque exhibited similar patterns and magnitudes, indicating that muscle torque primarily contributed to overall movement. However, at the distal joint, the elbow, interaction torque was often comparable in magnitude to the other torques, especially when contrasted with the shoulder. While there were no significant differences in the overall pattern between the two groups, the cerebellar ataxia group demonstrated greater inter-subject variation and less smooth torque curves

The relationship between the torques varied depending on location of the target. Differences between control and cerebellar groups were less obvious, and differences between the groups were not visually apparent. Focusing on the interaction between interaction torque and the other torques, it was observed that for targets T6 and T7—where the shoulder and elbow joints moved in opposite directions—interaction torque in both joints exhibited a correlated relationship with the other torques. This means that, for these two targets, interaction torque formed in a direction that assisted the overall movement represented by net torque and the movement effort shown by muscle torque.

#### Significant group differences were observed in the cross-correlation analysis between interaction torque and dynamic muscle torques during opposite movements.

**Figure 9.**
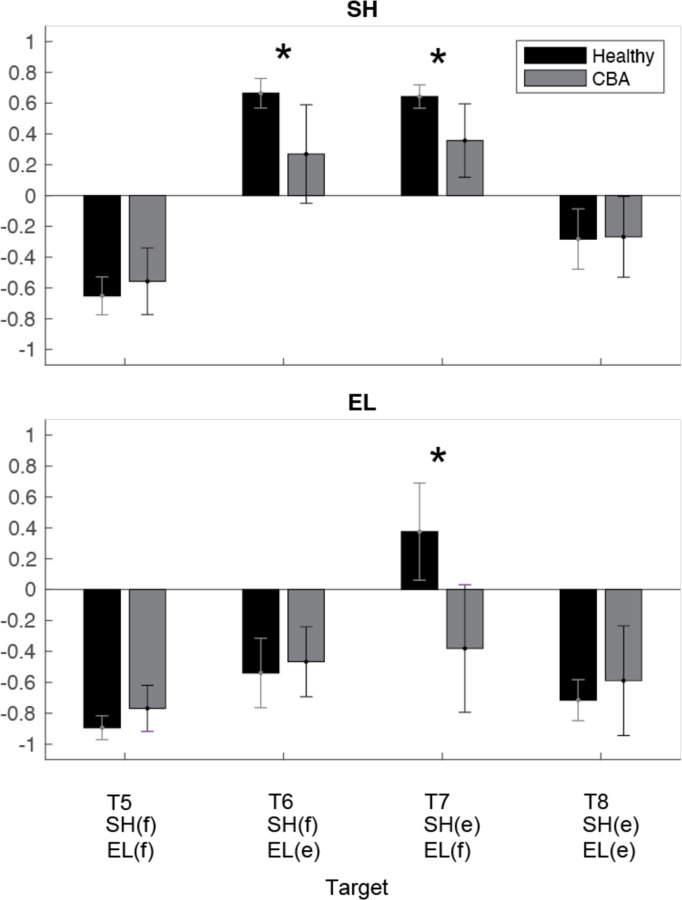
Zero-lag cross-correlation between dynamic muscle torque and interaction torque at shoulder and elbow joints for two-joint targets ( *: p<0.05.)

To quantitatively represent the relationship between the torques, we calculated the zero-lag cross-correlation between the dynamic muscle torque and the interaction torque to examine the simultaneous relationship between the torques. As shown in Figure 9, The cross-correlation values varied depending on the target location and joint movement combination. For the shoulder joint, a positive correlation between interaction torque and dynamic muscle torque was observed at targets 6 and 7, where the elbow and shoulder moved in opposite directions, suggesting that the interaction torque aided shoulder movement. In contrast, at targets 5 and 8, the interaction torque appeared to hinder shoulder movement. These findings are consistent with the torque patterns shown in Figure 8. For the elbow joint, the interaction torque generally acted in a way that hindered elbow movement. However, in the control group's movement toward target 6, the relationship between the two showed a positive correlation, which was notably different from the cerebellar group’s results.

The differences between the groups were most apparent at targets 6 and 7, where the shoulder joint showed a positive cross-correlation between interaction torque and dynamic muscle torque. At these two targets, the healthy control group exhibited significantly higher cross-correlation values between shoulder interaction torque and dynamic muscle torque compared to the cerebellar group (T6: F([1,248] = 21.986, p < 0.001; T7: : F([1,248] = 11.466, p < 0.001). This indicates that the control group was able to more effectively utilize interaction torques to assist joint movement, particularly at targets requiring complex, opposite movements between the elbow and shoulder. A difference between the groups was also observed at the elbow joint for target 7 (F([1,248] = 67.346, p < 0.001), but this was not the case for target 6.

#### There was a significant reduction in interaction torque contributions during opposite direction multijoint movements made by cerebellar patients (targets 6 and 7).

**Figure 10.**
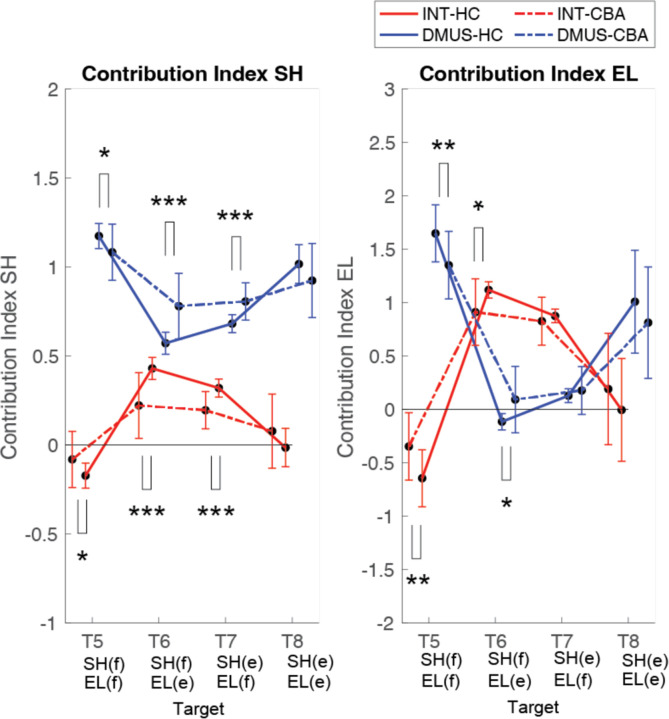
Contribution index. (*: p>0.05, **: p<0.01, and ***: p<0.001)

Figure 10 illustrates the contribution index, which represents the relative contribution of interaction torque and dynamic muscle torque to net torque. Figure 8 shows that the interaction torque tended to assist the net torque for both shoulder and elbow joints, during arm reaching toward Targets 6 and 7. This is indicated by positive contribution index values. In contrast, for Targets 5 and 8, the contribution index of the interaction torque was close to zero or relatively low, indicating a minimal influence of the interaction torque on the net torque. In the case of dynamic muscle torque, both joints showed relatively high positive contribution index values, demonstrating a significant contribution to the net torque for all two-joint targets. Given the overall slower arm reaching speed, it is expected that the influence of interaction torque would be smaller compared to dynamic muscle torque. However, during movements towards targets 6 and 7 at the elbow, the dynamic muscle torque approached zero, suggesting that interaction torque played a more dominant role in these movements.

Notably, the key difference between the two groups emerges at the shoulder joint for targets 6 and 7, where the interaction torque contributed positively to the net torque. Compared to the healthy control group, the cerebellar ataxia group displayed significantly reduced contribution of the interaction torque to the net torque at these targets, as shown by their lower contribution index. This suggests that the cerebellar group had difficulty utilizing interaction torque effectively to assist movement, leading to greater reliance on dynamic muscle torque, which may not fully compensate for the movement requirements of these complex, opposite tasks. (DMUS shoulder—T5: F[1, 33] = 4.689, p = 0.038, T6: F[1, 33] = 19.346, p < 0.001, T7: F[1, 33] = 19.229, p < 0.001; INT shoulder—T5: F[1, 33] = 4.689, p = 0.038, T6: F[1, 33] = 19.346, p < 0.001, T7: F[1, 33] = 19.229, p < 0.001; DMUS elbow— T5: F[1, 33] = 8.585, p = 0.006, T6: F[1, 33] = 7.108, p < 0.012; INT elbow— T5: F[1, 33] = 8.585, p = 0.006, T6: F[1, 33] = 7.108, p < 0.012).

## Discussion

Our findings revealed that the cerebellar ataxia group exhibited distinct differences in reaching kinematics and dynamics compared to the control group. The control group maintained near-straight hand trajectories across all targets, whereas the cerebellar ataxia group made curved trajectories that were greatest when shoulder and elbow joints had to move in *opposite direction*s (i.e., shoulder flexion and elbow extension (target 6) and vice-versa (target 7)). Kinematic simulations showed that the initial hand movement direction was highly sensitive to the relative elbow and shoulder onset times and joint velocities when the joints moved in *opposite direction*s. A small error in timing or velocity of joint movements led to misdirected hand motions early in movement. The typical pattern of initial movement incoordination was shoulder motion preceding elbow motion for these reaches.

In contrast, hand direction was relatively insensitive to joint motions that were in the *same* direction (i.e., both joints flexing (target 5) or extending (target 8)). When shoulder and elbow joints moved in the *same* direction, the cerebellar group made initial hand movements that were not statistically different from controls, though there was more variation between subjects in the cerebellar group. Kinematic simulations showed that changes in the relative onset time and joint velocities of elbow and shoulder did not affect these reaches as much. In sum, the cerebellar group struggled most on multi-joint movements when the joint velocities and onset times had to be under tight control.

Dynamic analysis revealed that when the joints move in *opposite direction*s, the interaction torque assisted the desired movement (i.e., the net torque) at both joints. In other words, the interaction torque correlated with the net torque, and the dynamic muscle torque was aided by the interaction torque to achieve the movement. Consistent with this, the control group showed a positive correlation between interaction and dynamic muscle torque at the shoulder, whereas the cerebellar group showed significantly less correlation. We calculated a contribution index which is the relative contribution of the interaction torque and dynamic muscle torque to the net torque. While the overall pattern was similar, the cerebellar group had reduced interaction torque contributions to the net torque at the shoulder joint, and most notably for targets 6 and 7.

Overall, this study highlights the increased sensitivity of hand movement to joint-relative motion and the assistive role of interaction torque in movements requiring opposing joint directions, with cerebellar ataxia patients struggled through altered joint onset delay and relative velocities. The significance of these findings lies in the deeper understanding they offer regarding motor coordination impairments in cerebellar ataxia, especially for tasks requiring complex inter-joint coordination. These insights can guide the development of more targeted rehabilitation strategies, aimed at improving motor control by addressing specific compensatory mechanisms, such as altered joint timing and velocity adjustments, that cerebellar ataxia patients employ to manage movement instability.

### The differences in hand path curvature depending on the target location cannot be explained by target distance alone.

In this study, the target locations are designed so that the combinations of joint movement direction and engagement required to reach them vary significantly, inherently making the arm-reaching movements toward each target heterogeneous. This variation in target locations results in differing demands on the shoulder and elbow joints. For example, reaching targets T6 and T7 requires opposite movements, whereas reaching T1 and T2 primarily involves elbow movements alone.

It is important to note that the distances to targets T6 and T7 (HC: mean 11.0cm, SD 0.9cm; CBA: mean 11.0 cm, SD 0.68cm) are not significantly different from the distances to targets T1 and T2 (HC: mean 13.8cm, SD 1.0cm; CBA: mean 13.7cm, SD 0.69cm). Despite the similarity in distances, the different joint motion combinations required for T6 and T7, involving both shoulder and elbow joints in opposite directions, contrast sharply with the simpler elbow-only movements required for T1 and T2. Additionally, while targets T5 and T8 also involve two-joint movements, these are same-direction multijoint movements, distinguishing them from the opponent combinations observed with T6 and T7. These differences in joint motion combinations have a significant impact on the quality of arm-reaching movements, particularly in cerebellar patients. For instance, as depicted in Figure 3, variables related to hand path curvature, such as maximum deviation ratio and hand path ratio, display significant increases for cerebellar patients at targets T6 and T7, compared to the healthy control group.

### Increased hand path curvature and joint variability in cerebellar ataxia highlight the challenges of multi-joint coordination.

The results of this study highlight significant differences in the kinematics of reaching movements between individuals with cerebellar ataxia and healthy controls. Specifically, cerebellar ataxia patients exhibited greater variability in both hand path and joint movement trajectories, particularly for targets that required multi-joint coordination, such as those involving both shoulder and elbow movements. These findings align with previous studies that have shown that multi-joint movements are more challenging for individuals with cerebellar damage, likely due to deficits in coordinating the complex interaction torques between joints (Bastian et al. 1996; Gibo et al. 2013).

The cerebellar ataxia group demonstrated significantly more curved trajectories for certain targets, particularly when the shoulder and elbow had to move in opposite directions. This increased curvature likely indicates a disruption in feedforward motor planning, suggesting that cerebellar ataxia patients struggle to accurately predict and compensate for the dynamic forces generated during complex multi-joint movements. The fact that hand paths were relatively straight for the healthy control group, regardless of the target, underscores the cerebellum's critical role in maintaining smooth, coordinated movement in such tasks.

The kinematic simulations further revealed that hand movement errors were highly sensitive to variations in joint onset timing and relative joint velocities, particularly for movements requiring opposing joint actions. Targets 6 and 7, which required one joint to flex while the other extended, proved especially challenging for cerebellar ataxia patients, who exhibited larger deviations from the target direction. These findings suggest that the coordination of joint movements, especially the precise timing of shoulder and elbow actions, is impaired in cerebellar ataxia, contributing to the characteristic movement inaccuracies observed in these individuals.

The variability observed in joint angle trajectories, particularly at the elbow, further supports the notion that cerebellar ataxia patients exhibit deficits in multi-joint coordination. The greater standard deviation of joint angles in the cerebellar group, as well as the larger range of onset time and joint change rate differences, reflects the difficulty these patients face in synchronizing their shoulder and elbow movements. This increased variability may be a compensatory mechanism employed by cerebellar ataxia patients to cope with the instability caused by impaired coordination, but it ultimately leads to less efficient and less accurate reaching movements.

These kinematic findings have important implications for rehabilitation strategies. Therapies aimed at improving joint coordination, particularly by focusing on the timing and velocity of multi-joint movements, may help reduce the movement errors observed in cerebellar ataxia. Additionally, training programs that target specific movement patterns involving opposing joint actions, such as those seen in Targets 6 and 7, could be particularly beneficial. By improving the ability of patients to anticipate and compensate for interaction torques, rehabilitation strategies could enhance motor function and reduce the movement variability seen in everyday tasks.

### Cerebellar ataxia alters hand movement dynamics and reduces torque coordination during opposing joint movements.

The phenomenon of individuals with cerebellar damage exhibiting different hand path patterns depending on the target location has been reported in several previous studies (Gibo et al. 2013; Zimmet et al. 2019), but Gibo et al. (2013) made notable contributions to the understanding of movement dynamics. In their horizontal arm reaching experiments, they noted that the hand path curvature of cerebellar patients varied depending on the target location, with significant curvature measured for targets requiring opposing movements of the elbow and shoulder. The authors suggested that this was due to the patients' failure to generate the appropriate torque necessary to create a straight hand path. This was attributed to the patients' inability to generate the appropriate torque needed to maintain a straight hand path. Consequently, a primary objective of this study was to examine the arm reaching kinematics and dynamics for each target location.

The experimental results of this study showed that, when shoulder and elbow movements are in opposite directions during hand reaching, individuals with cerebellar ataxia exhibited different hand movement patterns compared to the healthy control group. This difference was associated with changes in the onset time between the two joints or variations in the relative velocity of joint movements. Consequently, the movement dynamics of the cerebellar ataxia group demonstrated significantly lower values of joint torque correlation compared to the healthy control group. Additionally, the contribution of interaction torque to net torque was also decreased while reliance on dynamic muscle torque simultaneously increased in the cerebellar ataxia group. This aligns with previous research (Bastian et al. 2000) that suggests the arm coordination issues in cerebellar ataxia subjects stem not from an inability to generate adequate levels of phasic torque, but from improper compensation for interaction torque. However, this study offers more advanced findings, considering the effects of target location and providing deeper insights into both the kinematic and dynamic aspects.

The results from both kinematic and dynamic analyses demonstrate a clear connection between coordination deficits in cerebellar ataxia and the inability to manage interaction torques effectively during multi-joint movements. Kinematic data revealed increased variability in joint timing and velocity, leading to significant deviations in hand path, particularly in movements requiring opposing shoulder and elbow actions. This was most evident in target 6 and 7, where cerebellar ataxia patients exhibited substantial hand movement errors.

The dynamic analysis further clarified that these kinematic deviations were closely tied to the mismanagement of interaction torques. Controls were able to utilize assistive interaction torques and achieve smooth coordination across joints. In contrast, cerebellar patients showed a diminished ability to make use of supportive interaction torques, particularly in movements where shoulder and elbow joints had to move in opposite directions.

Together, the kinematic and dynamic results suggest that cerebellar ataxia patients’ movement inaccuracies stem not only from impaired motor planning (as indicated by the kinematic variability and deviated initial hand movement directions) but also from their inability to effectively harness the forces acting across joints. This inability to properly coordinate both kinematics and dynamics results in the observed erratic movement trajectories. Thus, rehabilitation strategies that focus on improving both the timing of joint movements and the ability to manage interaction torques could be particularly beneficial for cerebellar ataxia patients.

### Implications for future movement studies and the design of assistive devices for individuals with cerebellar ataxia.

For future studies on cerebellar ataxia, it is important to investigate multi-joint movements involving complex coordination between joints, rather than focusing solely on single-joint or single-target tasks. Studies limited to simpler movements do not reflect the broader challenges cerebellar patients face in daily activities. Instead, research should target more dynamic, multi-joint scenarios, such as reaching for various targets that require shoulder and elbow coordination in opposite directions. These tasks can expose deficits in interaction torque control, which significantly contributes to movement inaccuracy.

The insights gained from these studies can have profound implications for designing assistive devices. Current devices may not adequately support cerebellar patients’ specific motor deficits, as they fail to address issues of joint coordination and torque control in real-world scenarios. Future assistive technologies should incorporate real-time feedback mechanisms that help compensate for delayed or uncoordinated joint movements. For instance, wearable devices could be designed to monitor and adjust interaction torques, guiding patients toward smoother, more accurate movements by correcting misaligned joint speeds or delays.

## Figures and Tables

**Table 1.** Subject information

Group	HC	CBA
Headcount	17	16
Age	61.6 (±6.9)	60.0 (±11.9)
M/F	7/10	5/11
Body Weight (lbs)	164 (±26)	157 (±29)
UA segment length (cm)	31.7 (±2.6)	31.6 (±1.9)
LA segment length (cm)	39.8 (±2.8)	39.5 (±2.0)

**Table 2.** Characteristics of patients with cerebellar damage

Subjects	Age	Sex	Diagnosis	Total SARA (/40)	Arm-related SARA (/12)
CBA-01	69	M	SCA-27B ADCA3	3.5	1
CBA-02	69	F	SCA6	26	7
CBA-03	68	M	SCA6	26	7
CBA-04	50	F	SCA2	19	6.5
CBA-05	61	F	SCA6	29	7
CBA-06	68	F	EA2	13	4
CBA-07	42	F	SCA3	19	1.5
CBA-08	57	M	Sporadic, episodic	16.5	3
CBA-09	72	M	SCA5	24	7
CBA-10	50	F	SCA8	13	3.5
CBA-11	42	F	SCA3	24	8
CBA-12	54	F	Unknown etiology	13.5	2.5
CBA-13	80	F	SCA6	17	5
CBA-14	61	F	SCA5	30	8
CBA-15	73	F	SCA6	15.5	5
CBA-16	44	M	SCA8	14	4

**Table 3.** Inter-subject variations of the hand paths: Standard deviations of maximum deviation ratio

Group	Target							
	1	2	3	4	5	6	7	8
Control	0.0237	0.0423	0.0224	0.0228	0.0191	0.0437	0.0223	0.0174
Cerebellar	0.0296	0.0638	0.0453	0.0612	0.0373	0.0991	0.1444	0.0362

**Table 4.** Simulated vs experimentally measured hand deviation angles (deg ± standard deviation)

Group	Target			
	5	6	7	8
Heathy	−4.28 ± 4.46	11.56 ± 12.40	1.69 ± 7.55	10.49 ± 8.38
Cerebellar	−2.62 ± 12.10	8.94 ± 18.32	5.45 ± 18.56	15.79 ± 13.68
