# Supplementary material for: Cerebellar Reaching Ataxia is Exacerbated by Timing Demands and Assistive Interaction Torques
Source: bioRxiv. 2025 May 14:2024.10.28.620711. Originally published 2024 Oct 30. Preprint. [Version 7] doi: 10.1101/2024.10.28.620711 (PMC11565890; doi:10.1101/2024.10.28.620711)
Supplement: Supplement 1 [file media-1.docx]

**Supplementary Materials**


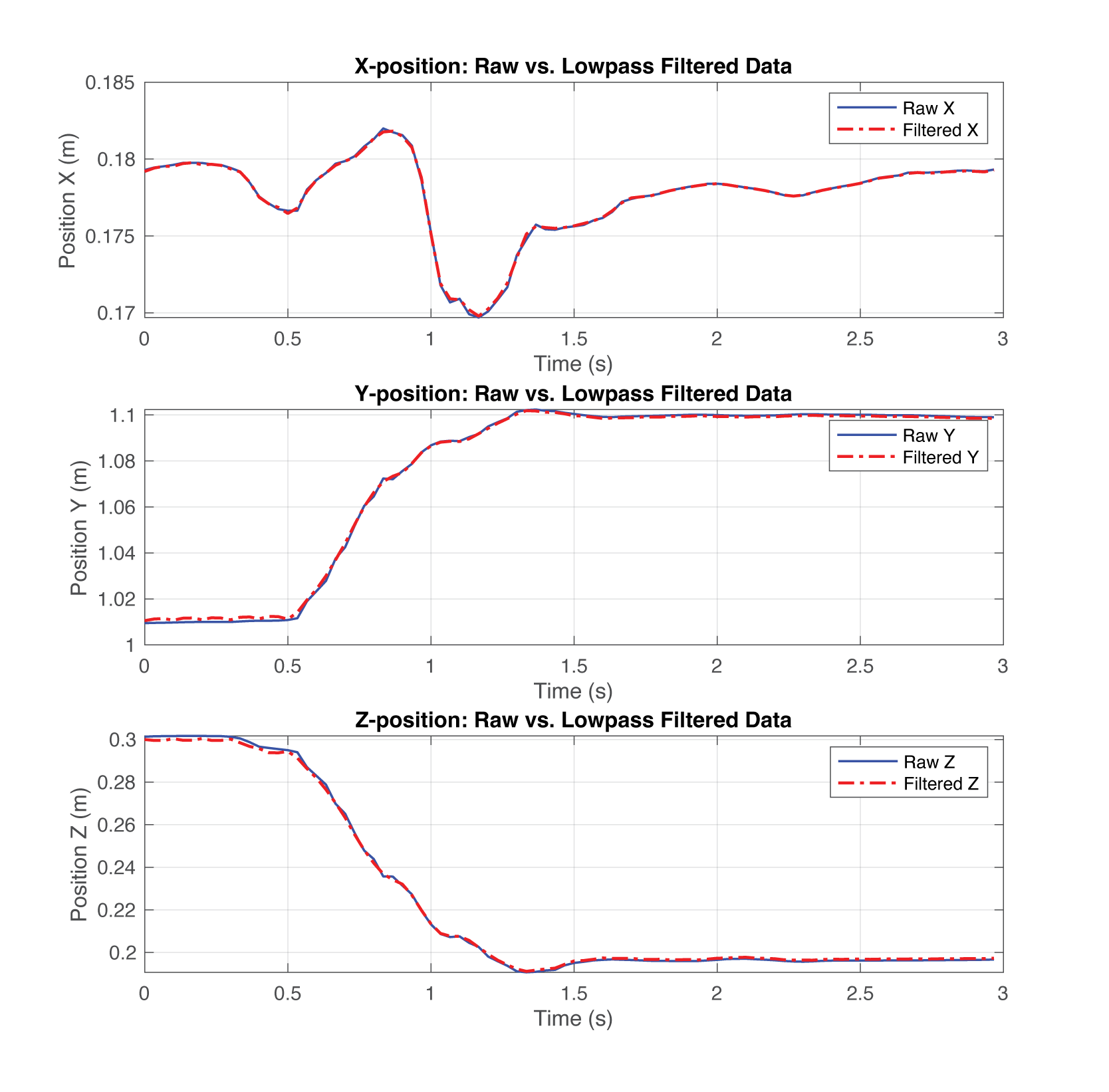


***Figure S1.*** *Raw and low-pass filtered hand position data (Control 7, Target 1, Trial 1). Hand position data in the x-, y-, and z-directions were low-pass filtered at 10 Hz using MATLAB’s ‘lowpass’ function to reduce potential noise. Example data are shown for one representative trial.*
